# *Drosophila* Insulin-Like Peptides DILP2 and DILP5 Differentially Stimulate Cell Signaling and Glycogen Phosphorylase to Regulate Longevity

**DOI:** 10.3389/fendo.2018.00245

**Published:** 2018-05-28

**Authors:** Stephanie Post, Galina Karashchuk, John D. Wade, Waseem Sajid, Pierre De Meyts, Marc Tatar

**Affiliations:** ^1^Department of Molecular Biology, Cell Biology and Biochemistry, Brown University, Providence, RI, United States; ^2^Department of Ecology and Evolutionary Biology, Brown University, Providence, RI, United States; ^3^Florey Institute of Neuroscience and Mental Health, University of Melbourne, Melbourne, VIC, Australia; ^4^School of Chemistry, University of Melbourne, Melbourne, VIC, Australia; ^5^LEO Pharma A/S, Ballerup, Denmark; ^6^Department of Cell Signalling, de Duve Institute, Brussels, Belgium; ^7^Department of Stem Cell Research Novo Nordisk A/S, Måløv, Denmark

**Keywords:** insulin, IGF, *Drosophila* insulin-like peptides, glycogen phosphorylase, glycogen, metabolism, aging, signaling bias

## Abstract

Insulin and IGF signaling (IIS) is a complex system that controls diverse processes including growth, development, metabolism, stress responses, and aging. *Drosophila melanogaster* IIS is propagated by eight *Drosophila* insulin-like peptides (DILPs), homologs of both mammalian insulin and IGFs, with various spatiotemporal expression patterns and functions. DILPs 1–7 are thought to act through a single *Drosophila* insulin/IGF receptor, InR, but it is unclear how the DILPs thereby mediate a range of physiological phenotypes. We determined the distinct cell signaling effects of DILP2 and DILP5 stimulation upon *Drosophila* S2 cells. DILP2 and DILP5 induced similar transcriptional patterns but differed in signal transduction kinetics. DILP5 induced sustained phosphorylation of Akt, while DILP2 produced acute, transient Akt phosphorylation. Accordingly, we used phosphoproteomic analysis to identify distinct patterns of non-genomic signaling induced by DILP2 and DILP5. Across all treatments and replicates, 5,250 unique phosphopeptides were identified, representing 1,575 proteins. Among these peptides, DILP2, but not DILP5, dephosphorylated Ser15 on glycogen phosphorylase (GlyP), and DILP2, but not DILP5, was subsequently shown to repress enzymatic GlyP activity in S2 cells. The functional consequences of this difference were evaluated in adult *Drosophila dilp* mutants: *dilp2* null adults have elevated GlyP enzymatic activity relative to wild type, while *dilp5* mutants have reduced GlyP activity. In flies with intact insulin genes, *GlyP* overexpression extended lifespan in a Ser15 phosphorylation-dependent manner. In *dilp2* mutants, that are otherwise long-lived, longevity was repressed by expression of phosphonull *GlyP* that is enzymatically inactive. Overall, DILP2, unlike DILP5, signals to affect longevity in part through its control of phosphorylation to deactivate glycogen phosphorylase, a central modulator of glycogen storage and gluconeogenesis.

## Introduction

Insulin and insulin-like growth factor signaling (IIS) is an extensive network crucial for development, growth, nutrient sensing, aging, and stress responses (1–3). Dysfunction in IIS contributes to metabolic syndrome, diabetes, and cancer (4, 5), yet genetic modification of IIS can extend lifespan in many animals (6–8). Mammalian insulin and IGF ligands each have respective receptors, although each ligand can activate either receptor, receptors can form hybrid dimers, and the receptors themselves activate similar kinase cascades with multiple redundant components (9). It is currently unknown how IIS ligands use common receptors and pathways to produce different cellular and organism phenotypes such as glucose homeostasis for insulin and development and differentiation for IGF. Here, we use *Drosophila melanogaster* as a model system to understand how various insulin-like peptides [*Drosophila* insulin-like peptides (DILPs)] function through the fly’s sole insulin/IGF tyrosine kinase receptor (InR) to mediate specific physiological traits.

In *Drosophila*, ligand-activated InR phosphorylates a single insulin receptor substrate (IRS) *chico*, the homolog of mammalian IRS1–4, to induce the phosphorylation cascade of phosphoinositide-3-kinase (PI3K), phosphoinositide-dependent-kinase-1, and Akt (protein kinase B) (10). This signal transduction culminates to repress the forkhead transcription factor dFOXO, the homolog of mammalian FOXO1, 3a, and 4 (11). Fly IIS and FOXO mutants affect larval growth, adult size, lipid metabolism, stress responses and aging (8, 12, 13). Data suggest that DILPs have distinct spatial and temporal expression patterns and regulate shared and specific functions (13, 14). For instance, embryos express *dilp4*, larvae express *dilp2, dilp3*, and *dilp5*, and pupae upregulate *dilp1* and *dilp6* expression (1, 13, 14). In adults, *dilp2* modulates adult lifespan and blood sugar (8, 15), *dilp5* mediates protein metabolism (16), and *dilp3* is suggested to regulate lipid metabolism (17). Given the diversity of these functions, it is poorly understood how specificity can be produced by similar ligands signaling through a common InR receptor.

The unique spatiotemporal expression patterns of DILPs may be sufficient to confer their specific phenotypes. An alternative, but not mutually exclusive model proposes that DILPs differentially activate InR to induce distinct cell signaling patterns that communicate specific downstream phenotypes. Studies from mammalian systems support such a signaling bias model: cells engineered to express only the insulin receptor (IR), IGF-1R, or IGF-2R produce distinct signaling and gene expression patterns in response to insulin, IGF-1, or IGF-2 (18, 19). At a biochemical level, Cieniewicz et al. (20) found that the IR was differentially phosphorylated and dephosphorylated on several individual tyrosine residues when cells were treated with insulin, IGF-1 or IGF-2. Furthermore, the IR but not IGF receptors regulates FKHR phosphorylation, GSK-3 inactivation, and glycogen synthesis (21, 22).

Models of ligand–receptor interaction propose that IR ligands with different receptor-binding kinetics can induce distinct downstream signaling (23, 24). Empirically, Sciacca et al. (25) found that the insulin analogs aspart, lispro, and glulisine produce more sustained Akt phosphorylation than insulin, which generates transient Akt phosphorylation. Analogs such as [B-Asp^10^] insulin and [A-His^8^, B-His^4^, B-Glu^10^, B-His^27^] insulin, which have strong receptor-binding affinity, induced sustained receptor phosphorylation and increased mitogenicity (26), and insulin analogs with weaker receptor-binding affinity produced less mitogenic potential (27, 28). Recently, decreased mitogenicity was reported for the insulin analog dicarba where the A6-A11 disulfide bond is replaced with a rigid bridge to reduce flexibility in its receptor interaction (29). Collectively, these data suggest that insulin, IGF, and insulin analogs can differentially bind to receptors to produce distinct signals and generate different downstream phenotypes.

*Drosophila* insulin-like peptide 2 and DILP5 are two principal hormones made in the insulin-producing cells of the adult *Drosophila* brain (30). They are thought to regulate sugar and protein metabolism respectively (31), and to uniquely respond to dietary sugar and protein (32). We compared cells stimulated *in vitro* with DILP2 or DILP5 to determine their cell signaling outputs measured as Akt and InR phosphorylation, transcript profiles and global patterns of protein phosphorylation. In S2 cells, DILP2 and DILP5 regulate highly similar sets of mRNA transcripts and are equally potent in their ability to stimulate Akt phosphorylation. On the other hand, at a given dose DILP2 and DILP5 differentially modulate the kinetics of Akt phosphorylation, with DILP2 producing acute stimulation and DILP5 generating prolonged signal. Expanding this non-genomic view of biased signaling, we used global phosphoproteomic analysis and found substantial qualitative differences between DILP2 and DILP5. In particular, DILP2 uniquely regulates dephosphorylation of glycogen phosphorylase (GlyP) at Ser15, the residue that represses enzymatic activity in mammals and *Drosophila* (33–35). In concordance, we find that *dilp2* mutant flies have elevated GlyP enzymatic activity, and that overexpression of *GlyP* extended longevity in adults with normal insulin in a GlyP phosphorylation-dependent manner that corresponded with GlyP enzymatic activity. In a complementary way, we infer that the phosphorylation status and enzymatic activity of *GlyP* contribute to the lifespan extension of *dilp2* mutants because expression of phosphonull, inactive *GlyP* partially rescues extended longevity of *dilp2* mutants. Together, these data demonstrate that non-genomic signaling bias at the cellular level plays a role in differential physiological and life history functions of two insulin-like peptides.

## Materials and Methods

### Cell Culture and DILP Stimulation

S2 cells were cultured in Schneider’s media (Gibco) with 10% FBS (Gibco) and maintained at 28°C with ambient CO_2_. For overnight serum depletion, cells were held for 16 h in Schneider’s media without FBS, supplemented with 0.5% BSA and 25 mM HEPES. DILP5 peptide (36) stock at 50 μM and DILP2 peptide stock (37) at 100 μM stimulated overnight depleted cells compared with control depleted cells treated in parallel with an equal volume of 0.001 N HCl solvent (vehicle). The dose and duration of DILP stimulation for each experiment is detailed in the figure legend.

### RNA-Seq and Data Analysis

S2 cells at a density of ~1 × 10^7^ cells per 100 cm cell culture dish were serum depleted overnight and stimulated with 100 nM DILP2, 100 nM DILP5 or vehicle for 1 h. Cells were washed with sterile, cold PBS and resuspended in lysis buffer (NE BioLabs magnetic mRNA isolation kit # S1550S). mRNA was purified and fragmented (Ambion # AM8740), then reverse transcribed to cDNA (Invitrogen SuperScript III # 18080051). DNA was purified by Agencourt Ampure XP beads and quantified by Qubit. DNA was repaired by End-It DNA Repair Kit (Epicenter, # ER0720) then supplemented with Klenow fragment (NE BioLabs # M0212s). Adapters were ligated using the T4 DNA Ligase Quick Ligase Kit (Enzymatics L603-HC-L) and PCR enriched using Phusion DNA polymerase (NE BioLabs # F-531). Libraries were gel purified, selecting DNA from 200 to 500 bp, and gel extracted (Qiagen Qiaquick # 28704). The selected libraries were sequenced on a HiSeq2500 with 50 bp single-end reads. Fastq files were assembled by Tophat and Bowtie, and assemblies were mapped to *D. melanogaster* dm3 assembly. In R, transcript counts and RPKM values were computed by the “easyRNASeq” package, and differential expression was calculated by the “edgeR” package. Transcript abundances by RPKM were trimmed to exclude genes with 0 counts to calculate coefficient of variance and determine the top 10% of genes with most variance. All raw RPKM expression data are listed in Table S1 in Supplementary Material.

### Quantitative RT-PCR

Adult flies, serum-depleted S2 cells, and DILP-treated S2 cells at a density of about 1 × 10^6^ cells/ml were lysed in Trizol (Invitrogen) by mechanical force with two 3.2 mm steel beads in a Tissuelyser. RNA was Trizol extracted, treated with Turbo DNase (Invitrogen), and quantified on a NanoDrop ND-1000 (Thermo Fisher Scientific Inc.). RNA was reverse transcribed with iScript cDNA synthesis (Bio-Rad Laboratories, Inc.). Quantitative RT-PCR was conducted on an ABI prism 7300 Sequence Detection System (Applied Biosystems) using SYBR green PCR master mix (Applied Biosystems). Relative mRNA levels were calculated relative to RP49 expression by the comparative Ct method. Primer sequences are listed in Table S2 in Supplementary Material.

### Western Blots

S2 cells at a density of about 1 × 10^6^ cells/ml were serum-depleted overnight and stimulated with DILP2, DILP5 or vehicle at the indicated concentration for the designated duration. Cells were washed briefly with sterile, cold PBS and resuspended in NP40 lysis buffer (Thermo Scientific # FNN0021) supplemented with 1 mM PMSF, PhosSTOP phosphatase inhibitor cocktail (Roche # 04906837001) and Protease Inhibitor Cocktail (Invitrogen # 78425). Cells were incubated on ice for 30 min, and vortexed every 10 min. Cell lysates were spun down for 10 min at 13K rpm and the supernatant incubated at 70°C with gel loading buffer and reducing reagent. Samples were loaded onto SDS-PAGE gels (Invitrogen NuPAGE # NP0321) and run at 200 V for 45 min. Gels were transferred to nitrocellulose membrane (Whatman # 10401396) and blocked with 5% BSA in TBS-T for 1 h. Membranes were incubated with primary antibody diluted 1:1,000 in 5% BSA in TBS-T overnight at 4°C with gentle rocking. Antibodies for Westerns were purchased from Cell Signaling Technology: *Drosophila* phospho-Akt Ser505 (# 4054); Pan-Akt (# 4691); Pan-phospho-ERK1/2 Thr202/Tyr204 (# 4370); Pan-ERK (# 9102); *Drosophila* phospho-S6K (# 9209); Pan-S6K (# 2708); IGF-R Tyr1131 (# 3021); actin (# 4967), or from Phospho-Solutions: *Drosophila* phospho-Akt Thr342 (# p104-342). Blots were washed for 5 min three times in TBS-T and incubated in horseradish peroxidase conjugated anti-rabbit secondary antibody (Jackson Immunoresearch) diluted 1:5,000 in 1% BSA for 1 h at room temperature. Subsequently, blots were washed for 5 min three times in TBS-T and incubated with ECL reagent (Perkin Elmer # NEL121001EA). Final blots were imaged and volume densitometrically quantified with ImageLab (Bio-Rad).

### Phosphopeptides Sample Preparation and Enrichment for LC–MS/MS Analysis

Cell pellets were lysed in urea buffer (8 M urea, 1 mM sodium orthovanadate, 20 mM HEPES, 2.5 mM sodium pyrophosphate, 1 mM β-glycerophosphate, pH 8.0, 20 min, 4°C), sonicated and cleared by centrifugation (14,000 *g*, 15 min, 4°C). Protein concentration was measured (Pierce BCA Protein Assay, Thermo Fisher Scientific, IL, USA) and a total of 100 µg protein per sample was subjected to trypsin digestion. Tryptic peptides were desalted using C18 Sep-Pak plus cartridges (Waters, Milford, MA, USA) and lyophilized for 48 h to dryness. Phosphopeptides were enriched with Titansphere Phos-TiO tips (GL Sciences, Tokyo Japan) following the manufacturer’s protocol with modifications. We first prepared the condition buffers (containing TFA, CH_3_CN, and lactic acid) and elution buffers (1% NH_4_OH in water and 40% CH_3_CN). Then, the condition buffer was added to the Phos-TiO tips (centrifuge at 3,000 *g*, 22°C). After conditioning, desalted tryptic peptides from Jurkat total lysates (CD3/4 stimulated or unstimulated) were mixed with a synthetic phosphoserine standard (FQpSEEQQQTEDELQDK, AnaSpec, San Jose, CA, USA) at a ratio of 5 fmol standard: 1 µg sample. The mixture was loaded onto tips using centrifugation at 1,000 *g* at 22°C. After loading, the column was washed with condition buffers followed by elution buffers. Acetic acid was used to acidify TiO_2_-enriched samples, which were dried almost to completeness. The dried-eluted phosphopeptides were reconstituted in buffer A (0.1 M acetic acid) at a concentration of 1 µg/µl, and 5 µl was injected for each analysis.

The LC–MS/MS was performed on a fully automated proteomic technology platform (38, 39) with an Agilent 1200 Series Quaternary HPLC system (Agilent Technologies, Santa Clara, CA, USA) connected to a Q Exactive Plus mass spectrometer (Thermo Fisher Scientific, Waltham, MA, USA). The LC–MS/MS workflow follows Ahsan et al. (40). Peptides were separated through a linear reversed-phase 90 min gradient from 0 to 40% buffer B (0.1 M acetic acid in acetonitrile) at a flow rate of 3 µl/min through a 3 µm 20 cm C18 column. Electrospray voltage of 2.0 kV was applied in a split flow configuration, and spectra were collected using a top-9 data-dependent method. Survey full scan MS spectra (*m*/*z* 400–1,800) were acquired at a resolution of 70,000 with an AGC target value of 3 × 10^6^ ions or a maximum ion injection time of 200 ms. The peptide fragmentation was performed *via* higher-energy collision dissociation with the energy set at 28 NCE. The MS/MS spectra were acquired at a resolution of 17,500, with a targeted value of 2 × 10^4^ ions or a maximum integration time of 200 ms. The ion selection abundance threshold was set at 8.0 × 10^2^ with charge state exclusion of unassigned and *z* = 1, or six to eight ions and dynamic exclusion time of 30 s.

### Phosphoproteomics Analysis

Peptide spectrum matching of MS/MS spectra of each file was searched against a species-specific databases (UniProt; downloaded 2/1/2015) using MASCOT v. 2.4 (Matrix Science, Ltd., London, UK). A concatenated database containing “target” and “decoy” sequences was used to estimate the false discovery rate (FDR) (41). Msconvert from ProteoWizard (v. 3.0.5047), using default parameters and the MS2Deisotope filter, was used to create peak lists for Mascot. The Mascot database search was performed with the following parameters: trypsin enzyme cleavage specificity, two possible missed cleavages, 10 ppm mass tolerance for precursor ions, 20 mmu mass tolerance for fragment ions. Search parameters permitted variable modification of methionine oxidation (+15.9949 Da) and static modification of carbamidomethylation (+57.0215 Da) on cysteine. To identify the phosphoresidues, we included additional variable modification of phosphorylation (+79.9663 Da) on serine, threonine, and tyrosine residues. The resulting peptide spectrum matches (PSMs) were reduced to sets of unique PSMs by eliminating lower scoring duplicates. To provide high confidence, the Mascot results were filtered for Mowse Score (>20). Peptide assignments from the database search were filtered down to a 1% FDR by a logistic spectral score as previously described (41, 42). To validate the position of the phosphorylation sites, the Ascore algorithm (43) was applied, and the reported phosphorylation site position reflected the top Ascore prediction.

### Relative Quantitation of Phosphopeptides

Phosphopeptide abundance was quantified from selected ion chromatograms (SIC) peak areas. Retention time alignment of individual replicate analyses was performed as described by Demirkan et al. (44). Peak areas were calculated from SICs in R 3.0 based on the Scripps Center for Metabolomics’ XCMS package (version 1.40.0). This approach performed multiple passes through XCMS’ central wavelet transformation algorithm (implemented in the centWave function) over increasingly narrower ranges of peak widths with parameters: mass window of 10 ppm, minimum peak widths ranging from 2 to 20 s, maximum peak width of 80 s, signal to noise threshold of 10 and detection of peak limits *via* descent on the non-transformed data enabled. SIC peak areas were determined for every phosphopeptide identified by MS/MS. In the case of a missing MS/MS for a particular peptide in one replicate, the SIC peak area was calculated according to the peptide’s isolated mass, and the retention time was calculated from retention time alignment. A minimum SIC peak area equivalent to the typical spectral noise level of 1,000 was required of all data reported for label-free quantitation. Individual SIC peak areas were normalized to the peak area of the standard phosphopeptide DRVpYHPF that was exogenously spiked before phosphopeptide enrichment and reversed-phase elution into the mass spectrometer. Quantitative analysis was applied to five replicate experiments. To select phosphopeptides that show a statistically significant change in abundance between control and treatment cells, *q*-values for multiple hypothesis tests were calculated based on *p*-values from two-tailed unpaired Student’s *t*-tests using the R package QVALUE as described by Storey (2003) and Storey and Tibshirani (45, 46).

### Glycogen and Glucose Quantification

Glycogen and glucose were quantified as described by Tennessen et al. (47). Flies were mated for 2 days, and females separated into groups of six flies. Food vials were changed every other day, and at age 8–10 days, flies were briefly anesthetized with light CO_2_, collected in a microcentrifuge tube and flash frozen in dry ice. With tubes kept on ice, flies were homogenized in 100 μl PBS using a motorized plastic pestle. A 10 μl aliquot was removed for BCA protein quantification, and the remaining 90 µl was heat treated at 70°C for 10 min. Samples were spun down at 14K rpm for 3 min at 4°C, and the supernatant removed to a new tube. Samples were diluted 1:10, and standard curve dilutions for glucose and glycogen were made by diluting stocks to 160 µg/ml, making 1:1 serial dilutions for 80, 40, 20, and 10 µg/ml. 25 µl of each sample was pipetted to four wells of a clear microplate, and 25 µl of each glucose or glycogen standard was pipetted to two wells. Amyloglucosidase enzyme (Sigma, # A1602) was diluted 1.5 µl into 998.5 µl PBS, and 25 µl diluted enzyme was pipetted to the glycogen standards and two sample wells. 25 µl PBS was pipetted to the glucose standards and to the other two sample wells. The plate was wrapped in parafilm and incubated at 37°C for 1 h. 100 µl Glucose Hexokinase Reagent (Thermo Scientific TR15421) was pipetted to each well, and the plate was incubated at room temperature with gentle rocking for 15 min. The absorbance was read at 340 nm on a SpectraMax M5 platereader using Softmax Pro software. The glycogen concentration was quantified by subtracting the glucose absorbance from the total glycogen + glucose absorbance and normalized to total protein content quantified by BCA.

### Glycogen Phosphorylase Activity Assay

Activity of glycogen phosphorylase was adapted and modified from protocols used for mammalian cells (48). Five female flies 7–10 days old were harvested in 150 µl NP40 lysis buffer with inhibitors (see Western Blots) without centrifugation. 10 μl lysate was combined with reaction mixture in a 96-well plate on ice. Reaction mixture consisted of 50 mM Na glycerophosphate pH 7.1, 10 mM K_2_PO_4_, 5 mM MgCl_2_, 1 mM DTT, 0.2% glycogen, 0.5 mM NAD^+^, 1.6 U phosphoglucomutase, and 1.6 U glucose-6-phosphate dehydrogenase in a total reaction of 300 µl. The plate was brought to room temperature (25°C), and fluorescence was measured at excitation 350 nm, emission 470 nm (SpectraMax M5 platereader) for NADH generation. Activity was calculated by determining the fluorescence after 45 min incubation, relative to total protein determined by BCA assay (Thermo Scientific # 23227).

### Cloning *Drosophila GlyP*

*GlyP* cDNA was obtained from the *Drosophila* Genomics Resource Center (stock # LD24485) and cloned into pENTR-TOPO Gateway vector (Thermo Fisher). Mutations S15A and S15D were made using the GENEART Site-Directed Mutagenesis System (Invitrogen A13282). Wild-type, S15A, and S15D *GlyP* coding sequences were sub-cloned into the *Drosophila* transgene vector pUAST-attB and were submitted to GenetiVision Corporation (Houston, TX, USA) for transgene embryo injection and stock generation.

### Fly Husbandry

Flies were reared and maintained at 25°C, 40% relative humidity and 12-h light/dark. Adults were maintained upon agar-based diet with cornmeal (0.8%), sugar (10%), and yeast (2.5%), or upon the same diet supplemented with 200 μM mifepristone (RU486) or ethanol control. Stocks were backcrossed to w^1118^ for at least five generations.

### Lifespan Assays

Two- to three-day-old mated female adult flies reared in density-controlled bottles were collected with light CO_2_ anesthesia and pooled in 1 l demography cages at a density of 100–125 flies per cage. Three independent cages were used per genotype. Food vials were changed every day for the first 3 weeks, then every 2 days for the remainder of the experiment. Dead flies were removed and recorded every other day. Survival analysis and Cox Proportional Hazard analysis were conducted in R using the “surv” package and “survdiff” function. To adjust for mortality caused by the RU486 covariate independent of genotype, Gompertz mortality models were fit to control genotypes given RU486 or ethanol vehicle using the “flexsurv” package and “flexsurvreg” function. The mortality estimate decreased by RU486 treatment was applied to Gompertz mortality models for test genotypes given RU486 or ethanol vehicle.

### Starvation Assays

Two- to three-day-old mated female adult flies reared in density-controlled bottles were collected with light CO_2_ anesthesia into glass vials containing 1% agar solution at a density of about 15 flies per vial. Eight independent vials were used per genotype. Vials were changed every 2–3 days to ensure flies did not desiccate. Dead flies were counted every 8 h. Data analyzed as in Section “Lifespan Assays.”

## Results

**Figure 1:**
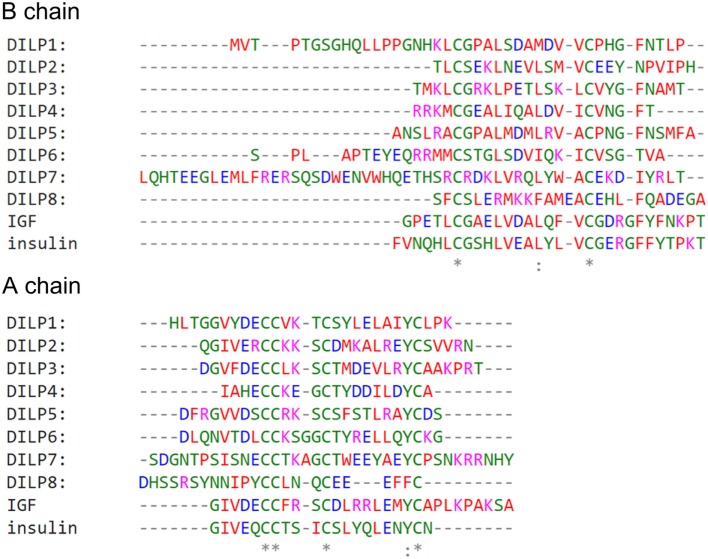
*Drosophila* insulin-like peptide (DILP) amino acid sequences have a low degree of identity between each other and with human insulin and IGF. DILP B chain and A chain sequences based on predicted cleavage sites. Alignment made using EMBL-EBI Clustal Omega (https://www.ebi.ac.uk/Tools/msa/clustalo/).

*Drosophila* insulin-like peptides 1–8 have varied physiological functions (8, 49, 50) which may arise from specific amino acid sequences and structures that interact with InR in precise ways. When DILP peptide sequences are aligned with that of human insulin and IGF (Figure 1), the B and A chains of the proposed mature pro-hormones show low sequence identity among DILPs and between DILPs, insulin and IGF, except for cysteine residues required for disulfide bonds connecting the B and A chains (51, 52). In addition, among most ligands there is a highly conserved tyrosine in the A chain and a leucine in the B chain that are proposed to function in ligand–receptor interactions (36). DILP7 and DILP1 are longer than other DILPs and have very unique sequences relative to DILPs 1–6. Human insulin, IGF, and some DILPs contain one or two phenylalanines at the C-terminal tail of the B chain, although these residues are not found in DILP2, DILP6 and DILP7.

**Figure 2:**
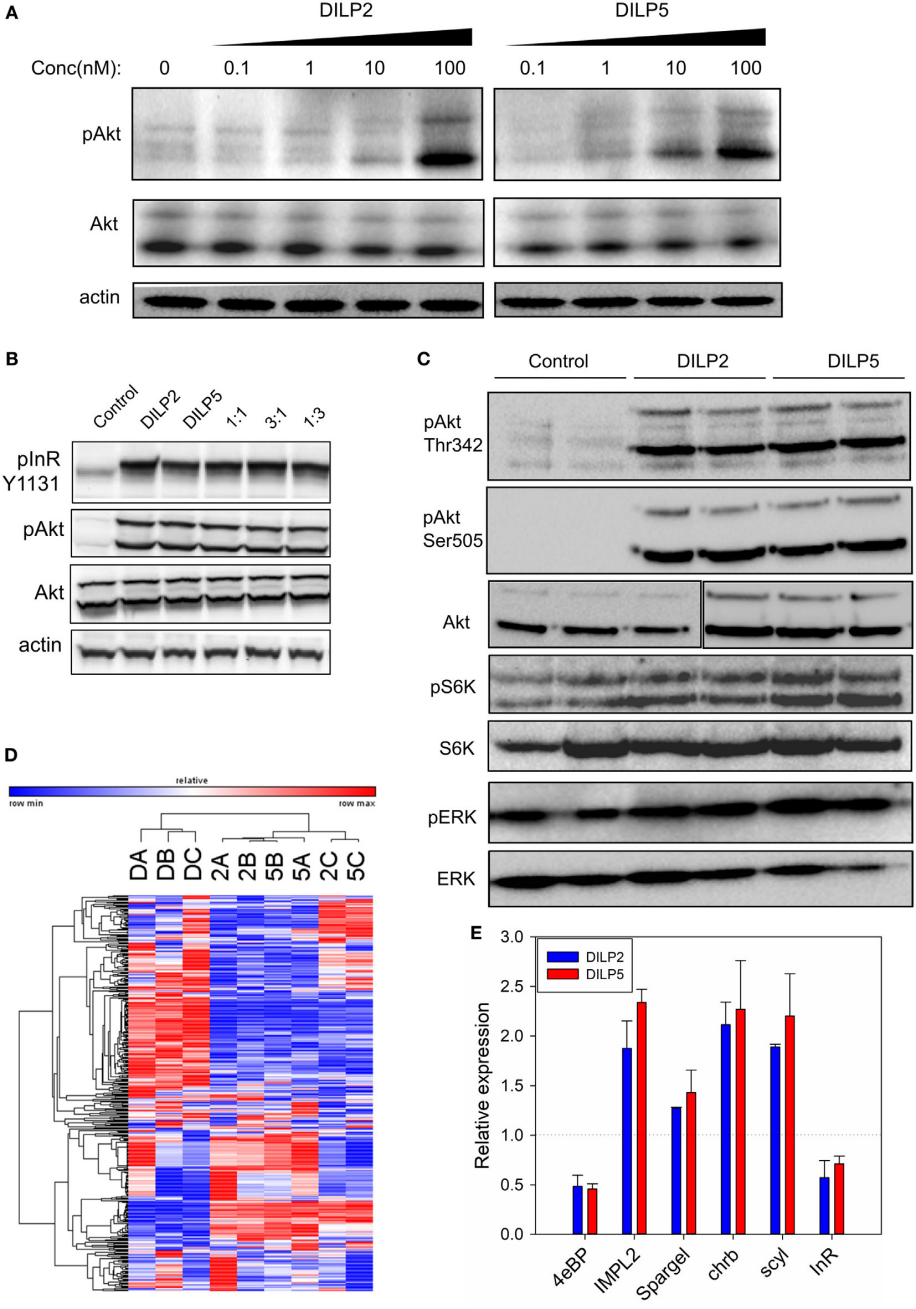
*Drosophila* insulin-like peptide (DILP) 2 and DILP5 induce similar signaling in S2 cells. **(A)** DILP2 and DILP5 stimulate Akt phosphorylation with comparable potency. S2 cells were stimulated with DILP2 or DILP5 for 5 min at the specified concentrations. **(B)** DILP2 and DILP5 activate InR and Akt the same in competition assays, inducing similar phosphorylation each alone, with equal parts each ligand or, with an excess of one ligand. S2 cells stimulated for 5 min with DILP2 or DILP5 at 100 nM, with 50 nM DILP2 and 50 nM DILP5 (“1:1”), with 75 nM DILP2 and 25 nM DILP5 (“1:3”), and with 25 nM DILP2 and 75 nM DILP5 (“1:3”). **(C)** DILP2 and DILP5 similarly stimulate pAkt, pS6K, and pERK at 100 nM for 5 min. **(D)** DILP2 and DILP5 at 100 nM for 1 h induce similar gene expression profiles. Heatmap represents the transcript RPKM values with the top 2.5% coefficient of variation between samples: DILP2 (“2”), DILP5 (“5”), or control serum-depletion (“D”). **(E)** DILP2 and DILP5 at 100 nM for 1 h induce similar changes in gene expression measured by qPCR.

*Drosophila* insulin-like peptide 2 and DILP5 are similarly expressed and released from brain medial secretory neurons in adult *Drosophila*, yet appear to have distinct roles in glucose and protein metabolism and in aging (8, 16). To understand how these related ligands generate different outcomes *in vivo*, we treated *Drosophila* S2 cells in culture with purified synthetic DILP2 (37) and recombinant DILP5 (36). At doses ranging from 0.1 to 100 nM, DILP2 and DILP5 stimulated comparable increases in Akt phosphorylation at Ser505 (Figure 2A), suggesting they have similar potency. Likewise, DILP2 and DILP5 were equally efficient at stimulating InR and Akt phosphorylation in a ligand competition assay (Figure 2B). Finally, DILP2 and DILP5 similarly stimulated phosphorylation of Akt at Thr342, S6K at Thr398, and ERK at Thr202/Tyr204, though S6K phosphorylation is slightly stronger after DILP5 stimulation than DILP2 (Figure 2C). Together, these data suggest that the synthetic DILP2 and recombinant DILP5 reagents have comparable potency and ability to stimulate S2 cell signaling.

Human insulin and IGF, and *Drosophila* DILPs regulate activity of FOXO transcription factors to affect downstream pathways through control of gene expression (53–55). To determine whether DILP2 and DILP5 stimulate different or similar gene expression profiles, we conducted RNA-Seq from mRNA of S2 cells stimulated with each DILP at 100 nM for 1 h (Figures 2D,E; Figure S1 and Table S1 in Supplementary Material). Transcript profiles induced by DILP2 and DILP5 were not distinguishable based on hierarchical clustering of the top 2.5% responding genes, while profiles stimulated by DILP2 and DILP5 were clearly distinct from profiles of cells in the absence of DILP (depletion control) (Figure 2D). On the other hand, DILP2, DILP5, and control profiles were mutually distinct based on principle component analysis of all RPKM values (Figure S1B in Supplementary Material). DILP5 stimulation induces and represses more genes overall than DILP2 stimulation. Of the 2,053 genes regulated by DILP5 and 1,646 genes regulated by DILP2 (differentially expressed relative to unstimulated controls, DE, FDR < 0.05), 1,366 genes were shared between DILP2 and DILP5 (Figure S1A in Supplementary Material; Table 1). Several of these shared, differentially expressed genes were validated by qRT-PCR from independent biological samples (Figure 2E). About 33% of DILP5 targets are unique to DILP5 and approximately 16% of DILP2 targets are unique to DILP2 (Table 1; Figure S1A in Supplementary Material). Two of the uniquely DILP2-regulated genes, Hex-C and CG33774, were validated by qRT-PCR from independent biological samples (Figure S1E in Supplementary Material).

**Table 1:** DE genes stimulated by *Drosophila* insulin-like peptide (DILP) 2 and DILP5: top 10 with statistical significance.

Comparison	Flybase ID	Gene	log2 Fold change	False discovery rate
DILP2 vs control	FBgn0036165	chrb	1.327375	4.38E−160
	FBgn0001257	ImpL2	1.267441	5.04E−158
	FBgn0261625	CG42708	1.172664	1.45E−144
	FBgn0041094	scyl	1.175456	7.91E−140
	FBgn0033391	CG8026	1.120938	3.63E−131
	FBgn0052103	CG32103	1.044493	2.74E−127
	FBgn0035542	CG11347	−1.15499	4.46E−109
	FBgn0001332	L	−1.20772	1.72E−108
	FBgn0023407	B4	0.815275	3.01E−96
	FBgn0015903	apt	1.088881	4.05E−91

DILP5 vs control	FBgn0036165	chrb	1.355795	9.66E−179
	FBgn0261625	CG42708	1.258132	7.79E−166
	FBgn0033391	CG8026	1.158735	5.35E−123
	FBgn0035542	CG11347	−1.21851	2.25E−118
	FBgn0052103	CG32103	1.037735	8.81E−114
	FBgn0015903	apt	1.175589	1.35E−112
	FBgn0041094	scyl	1.106372	4.55E−108
	FBgn0001257	ImpL2	1.340038	1.49E−103
	FBgn0001332	L	−1.26188	2.36E−98
	FBgn0259176	bun	−0.81646	1.84E−90

Unique to DILP2 vs control	FBgn0004893	bowl	−0.19777	0.003551
	FBgn0020440	Fak56D	−0.186	0.003782
	FBgn0038013	CG10038	−0.22335	0.008633
	FBgn0001149	GstD1	−0.22084	0.000232
	FBgn0034440	CG10073	−0.41936	0.019479
	FBgn0035710	SP1173	0.155922	0.014907
	FBgn0024177	zpg	0.24921	0.008772
	FBgn0002526	LanA	0.105225	0.0386
	FBgn0022699	D19B	−0.14368	0.033286
	FBgn0005672	spi	−0.12572	0.034756

Unique to DILP5 vs control	FBgn0032785	CG10026	−0.50202	0.016946
	FBgn0004635	rho	−0.2252	0.032889
	FBgn0037492	CG10050	0.427592	0.003207
	FBgn0038032	CG10096	−0.79401	0.008805
	FBgn0038033	CG10097	−0.78267	0.010873
	FBgn0003742	tra2	0.163187	0.039883
	FBgn0010638	Sec61beta	0.176851	0.021728
	FBgn0032799	CG10166	0.161421	0.015904
	FBgn0028554	xl6	0.157449	0.007431
	FBgn0037443	CG1021	0.141883	0.022384

DILP2 vs DILP5	FBgn0033865	CG6220	−0.40876	3.27E−06
	FBgn0053774	CG33774	−0.73463	0.001238
	FBgn0044510	mRpS5	−0.25787	0.044839

The log2 fold changes for genes regulated by either DILP were low overall: most were below twofold although some were up to fourfold for DILP2 stimulation and fivefold change for DILP5 (Figures S1C,D in Supplementary Material). Despite modest effect size, we observed low FDR for most differentially expressed genes (Table 1). However, only three genes showed statistical significance for differential expression between DILP2 and DILP5 (Table 1). Overall, DILP2 and DILP5 appear to have largely similar effects on the transcriptional responses of cells, at least on the time point of 1 h.

**Figure 3:**
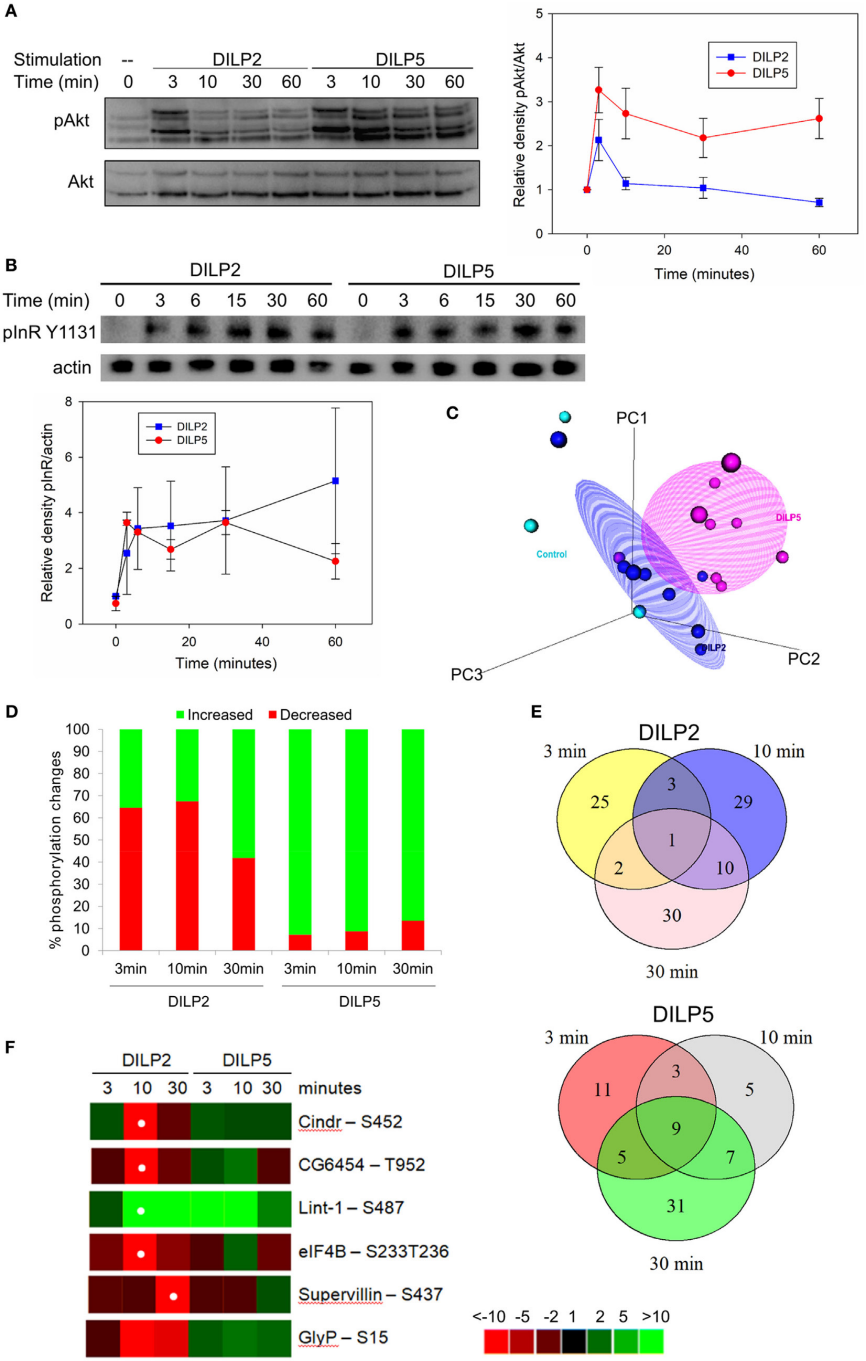
*Drosophila* insulin-like peptide (DILP) 2 and DILP5 stimulate distinct patterns of protein phosphorylation. **(A)** DILP2 and DILP5 at 100 nM stimulate distinct kinetics of phosphorylation upon Akt across 1 h. Representative blot (left) and quantification (right) of band densitometry analyzed in ImageLab (Bio-Rad), two-way ANOVA DILP2 vs DILP5 *p* < 0.001, *post hoc p* < 0.05 for *t* = 3 min, 10, 30, and 60 min. **(B)** DILP2 and DILP5 at 100 nM stimulate pInR to similar extents over 1 h. Representative blot (top) and quantification (bottom) two-way ANOVA *p* = 0.482. **(C)** PCA plot for phosphoproteomic data distinguishes among control, DILP2 stimulation, and DILP5 stimulation. **(D)** Phosphosites increased or decreased by DILP2 or DILP5 at 100 nM for the designated time points (cutoffs to call phosphosites inferred from data-driven fold-change threshold, see Figure S2 in Supplementary Material). **(E)** Venn diagram of phosphopeptides changed by DILP2 (top) and by DILP5 (bottom) across time. There are fewer overlapping phosphopeptides among DILP2 stimulation time points than among DILP5 stimulation time points. **(F)** Heatmap of phosphorylation events regulated by DILP2 and not by DILP5. White dot indicates false discovery rate (FDR) < 0.05 after adjustment for multiple comparison. Glycogen phosphorylase (GlyP) Ser15 FDR = 0.1.

In addition to transcription, insulin and IGFs modulate non-genomic cell signaling. In particular, human insulin and IGF ligands can induce specific cell responses by generating different kinetics of phosphorylation upon Akt and the insulin and IGF receptors (20, 25). To explore how DILP2 and DILP5 affect phosphorylation kinetics through a common receptor in *Drosophila*, we studied the time course of Akt phosphorylation in DILP2- and DILP5-stimulated cells by Western blots (Figure 3A). DILP2 rapidly and transiently induces pAkt, with a peak at 3 min that quickly recedes to baseline. On the other hand, DILP5 stimulates sustained pAkt for at least 1 h (Figure 3A). Such differences could arise from differences in negative feedback that determine signal termination or persistence as receptors are internalized and degraded, maintained in endosomes, or continue to signal from the cell surface (56, 57). To assess InR signaling persistence, we measured InR Tyr1131 phosphorylation over a 1 h time course of DILP2 or DILP5 stimulation. DILP2 and DILP5 stimulate similar, sustained receptor phosphorylation (Figure 3B), suggesting that dynamics of receptor activation alone does not account for the differential kinetics of pAkt in DILP2- and DILP5-stimulated cells.

Insulin and IGF stimulate phosphorylation on proteins besides Akt, including GSK3β, MAPK, and mTOR (58, 59). To more fully identify differences that are potentially induced by DILP2 and DILP5 signaling, we conducted an unbiased phosphoproteomic analysis in S2 cells treated with DILP2 or DILP5 at 100 nM for 3, 10, or 30 min (Table S3 in Supplementary Material). Among all samples and replicates, we detected 5,250 unique phosphosites corresponding to 1,575 proteins. Table 2 lists phosphosites significantly changed by DILP2 or DILP5 at individual time points compared with control. Phosphosites altered by DILP2 stimulation but not by DILP5 included Cindr (Ser452), CG6454 (Thr952), and Supervillin (Ser437) (Table 2; Figure 3F). Few phosphosites were significantly altered based on rigorous criteria (FDR < 0.05), likely because technical variation between biological replicates was high (Figure 3C). Nonetheless, principle component analysis reveals that combined time points of DILP2 and DILP5 phosphoproteomes separate from each other and relative to control cells in three dimensions. The first three principle components accounted for approximately 86.21% of the variance in the data. Time points at 3 min were more similar to control samples (data not shown); however, across all time points, DILP2 and DILP5 produce distinct global phosphorylation patterns (Figure 3C, MANOVA *p* = 0.005).

**Table 2:** Significantly altered phosphosites stimulated by *Drosophila* insulin-like peptide (DILP) 2 and DILP5.

Treatment	Protein	Phosphosite	Ratio	False discovery rate
DILP2, 10 min	slender lobes	Ser780	0.0249	0.0479
	daughter of sevenless	Thr515 Thr518	34.1037	0.0479
	sosondowah	Ser179	0.0218	0.0479
	germinal center kinase III	Ser454	0.0342	0.0479
	eIF4B	Ser233 Thr236	0.0014	0.0479
	CG6454	Thr952	0.0358	0.0479
	CG3680	Ser424	0.0107	0.0479
	CG1908	Ser487	197.1951	0.0479
	BtbVII	Thr243 Ser245	640.7984	0.0479
	Cindr	Ser452	0.0049	0.0479

DILP2, 30 min	CG32306	Ser437	0.0147	0.0479
	East	Ser467	0.0615	0.0479
	MAP3K/MLK	Ser582	6.9046	0.0479

DILP5, 3 min	TRAM	Ser359	5.8001	0.0479
	Pez	Ser760 Ser763	24.6908	0.0479

DILP5, 30 min	sterile 20-like kinase	Ser357	302.1428	0.0479

To further resolve potential differences between the DILP2- and DILP5-stimulated phosphoproteomes, we calculated the inflection point for all phosphopeptide fold changes in each condition as previously described (44, 60). The inflection points assigned conservative fold-change thresholds as criteria for altered phosphosites (Figure S2 and Table S4 in Supplementary Material). Over the time course, DILP5 largely increases phosphorylation at the detected phosphosites, while DILP2 equally increases or decreases phosphorylation (Figure 3D). In addition, the majority phosphosites changed by DILP2 were unique to one time point, but many of the phosphosites affected by DILP5 were observed across two or three time points (Figure 3E), consistent with the patterns of pAkt kinetics we observed by Western blot.

Among several phosphorylation events unique to DILP2 or DILP5 by inflection point analysis (Figure S2 and Table S4 in Supplementary Material), GlyP (Ser15) was greatly decreased in abundance by DILP2 but not by DILP5 (Figure 3F). Glycogen phosphorylase (GlyP) is notable as a conserved glycogen catabolic enzyme and the rate-limiting step in glycogenolysis (35, 48, 61, 62), while glycogen storage is emerging as a potential mediator of aging in several model systems (63, 64). The activity of GlyP is regulated through several mechanisms, including phosphorylation of Ser14 in mammalian phosphorylase (65), the analogous residue to *Drosophila* GlyP Ser15. Phosphorylation of Ser14 activates GlyP, and mammalian insulin represses this posttranslational modification (65). Our result shows that DILP2, but not DILP5, leads to dephosphorylation of GlyP at Ser15, suggesting that DILP2 specifically inactivates this central enzyme of glucose metabolism.

**Figure 4:**
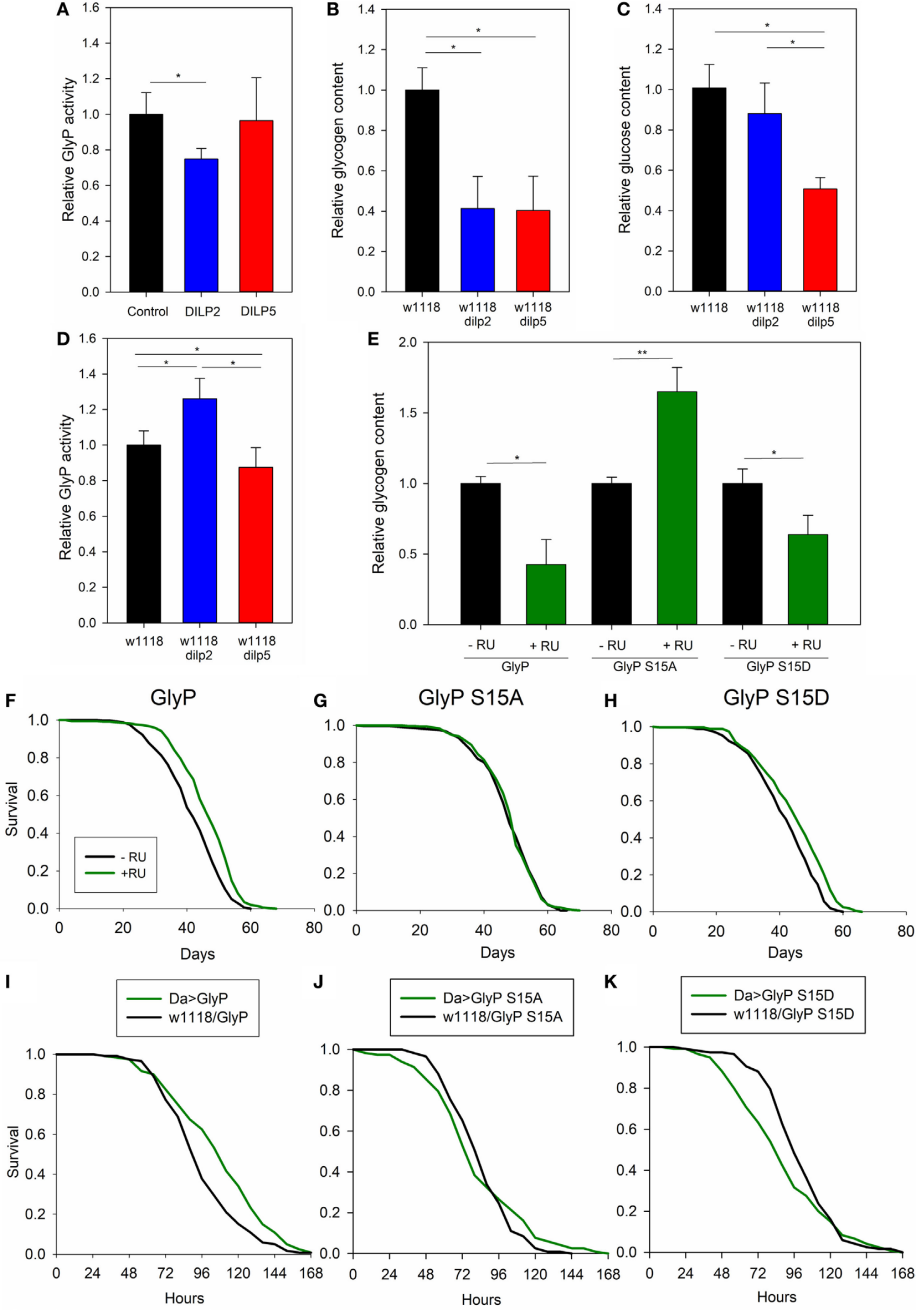
*Drosophila* insulin-like peptide (DILP) 2 and DILP5 differentially regulate glycogen phosphorylase (GlyP) to modulate lifespan and physiology. **(A)** DILP2 represses GlyP activity in S2 cells relative to cells in control conditions or those stimulated by DILP5. Serum-depleted S2 cells treated with 100 nM DILP or vehicle for 15 min. ANOVA *p* = 0.032, *n* = 6. **(B,C)** *dilp2* and *dilp5* mutants have decreased glycogen content, and *dilp5* mutants have decreased glucose content, relative to wild type. Each replicate from six female flies, age 10 days post-eclosion and 8–10 biological replicates. Glycogen **(B)** ANOVA *p* < 0.001 and glucose **(C)** ANOVA *p* < 0.001. **(D)** *dilp2* mutants have increased GlyP activity relative to wild type; *dilp5* mutants have decreased activity. Each replicate from 6 female flies, age 10 days post-eclosion, 11 biological replicates, ANOVA, *p* < 0.001. **(E)** Whole animal glycogen is affected by overexpressing GlyP with the DaGS-Gal4 driver as a function of Ser15. Each replicate, six female flies, age 10 days post-eclosion, four to five biological replicates. **(F–H)** Systemic GlyP overexpression extends longevity in a Ser15 dependent manner. DaGS-Gal4 (RU486 inducible) drove: **(F)** wild-type GlyP (Cox hazard analysis, χ^2^ = 46.5, *p* < 0.0001), **(G)** phosphonull GlyP S15A (Cox hazard analysis, χ^2^ = 0.1, *p* = 0.75), **(H)** phosphomimetic GlyP S15D (Cox hazard analysis, χ^2^ = 30.5, *p* < 0.0001). Each genotype cohort *n* = 348–366 adults. **(I–K)** Systemic GlyP overexpression regulates starvation resistance in a Ser15 dependent manner. Da-Gal4 drives: **(I)** GlyP (Cox hazard analysis, χ^2^ = 10.3, *p* = 0.001), **(J)** phosphonull GlyP S15A (Cox hazard analysis, χ^2^ = 0.2, *p* = 0.68), **(K)** phosphomimetic GlyP S15D (Cox hazard analysis, χ^2^ = 4, *p* = 0.05). Each genotype cohort *n* = 115–120 adults.

We conducted metabolic studies to verify that DILP2 specifically modulates glycogen metabolism and GlyP activity. First, DILP2 stimulation decreased GlyP enzymatic activity in S2 cells while DILP5 stimulation did not (Figure 4A). As well, measured from whole adults, GlyP activity was elevated in *dilp2* mutants but not in *dilp5* mutants (Figure 4D). Second, while *dilp2* and *dilp5* mutants showed reduced total glycogen content compared with wild-type flies (Figure 4B), *dilp5* mutants also have less total glucose (Figure 4C), suggesting that *dilp2* and *dilp5* mutants differentially modulate glycogen turnover. DILP2 appears to repress GlyP activity, and mutation of *dilp2* permits catabolism of glycogen to maintain glucose titers. DILP5 does not directly modulate GlyP activity, and mutation of *dilp5* must affect glycogen and glucose levels through an alternative metabolic pathway.

Unique among *Drosophila* IIS ligands, mutation of *dilp2* is sufficient to extend longevity, and reduced *dilp2* expression is consistently observed in various IIS manipulations that slow aging (8, 15, 53). Here, we report that reduced *dilp2* activates GlyP. Notably, chronological lifespan is reduced in yeast mutant for glycogen phosphorylase (64), and lifespan is extended in *Caenorhabditis elegans* deficient in glycogen synthesis (63). Therefore, we tested whether *GlyP* overexpression extends *Drosophila* lifespan. Wild-type *GlyP, GlyP* phosphonull (S15A, inactive enzyme), and *GlyP* phosphomimetic (S15D, constitutively active) were overexpressed using a systemic, RU486-inducible driver to express transgenes exclusively in adults and to provide exact coisogenic controls. Wild-type *GlyP* (Figure 4F) and constitutively active *GlyP* (S15D) (Figure 4H) extended lifespan. Expression of inactive *GlyP* (S15A) (Figure 4G) had no effect on survival. These data suggest that *Drosophila* lifespan is modulated by Ser15 phosphorylation of *GlyP* in response to DILP2. As well, many long-lived IIS mutants are resistant to starvation, and we found that expression of wild-type *GlyP* similarly improved starvation survival (Figure 4I). Starvation survival was not improved by phosphonull *GlyP* (Figure 4J), while phosphomimetic *GlyP* reduced resistance to starvation (Figure 4K). As noted, a recent study in *C. elegans* revealed that decreased glycogen increased longevity (63). Here, *Drosophila* longevity is increased by expressing wild-type or constitutively active *GlyP*, where each decreases total glycogen. Flies that express a phosphonull S15A *GlyP* have normal lifespan and increased total glycogen (Figure 4E).

**Figure 5:**
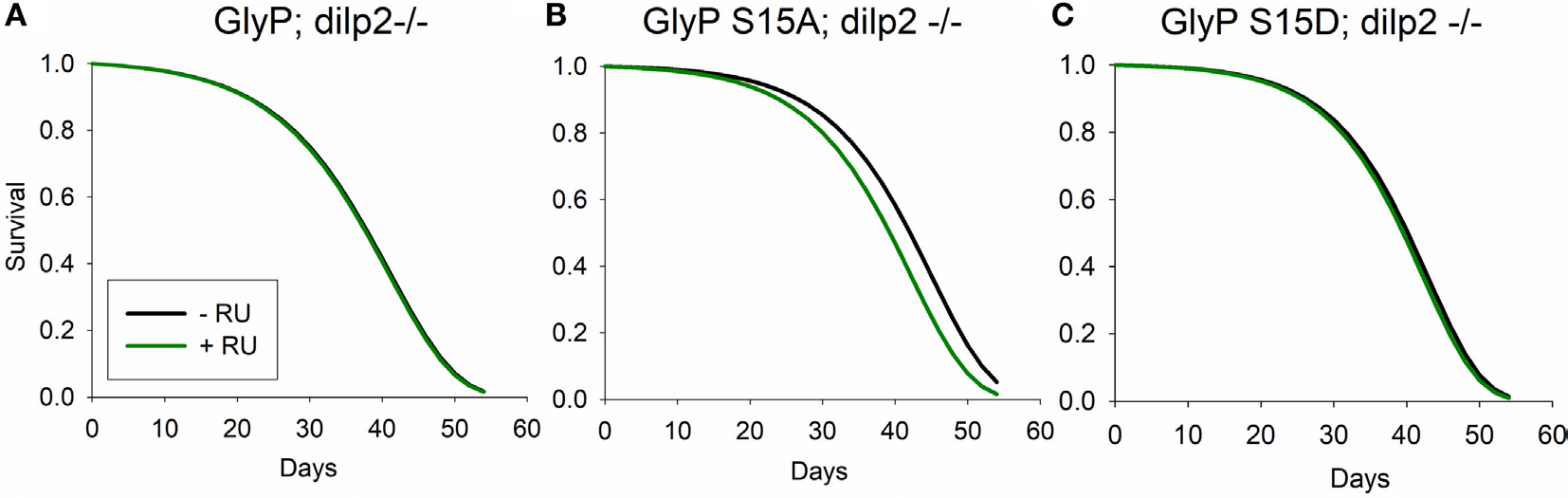
Glycogen phosphorylase (GlyP) phosphorylation is required for longevity conferred by mutation of *dilp2*. Survival calculated from life tables based on estimated Gompertz mortality parameters for UAS-GlyP expression in a *dilp2* mutant background with transgenes driven by systemic DaGS-Gal4. Gompertz mortality scale (l, frailty) parameters of transgene expression genotypes were adjusted for the impact of RU alone upon mortality estimated from control genotypes. **(A)** Overexpression of wild-type GlyP does not extend longevity (Cox hazard analysis, χ^2^ = 0.1, *p* = 0.76), **(B)** expression of GlyP S15A decreases lifespan (Cox hazard analysis, χ^2^ = 24.2, *p* < 0.0001), and **(C)** expression of GlyP S15D does not extend longevity (Cox hazard analysis, χ^2^ = 1.2, *p* = 0.28). Each genotype cohort *n* = 351–361 adults.

Our data suggest that mutation of *dilp2* modulates lifespan in part through non-genomic regulation of *GlyP*. To directly test this hypothesis, we conducted genetic epistasis analysis. With a systemic, RU486-inducible Gal4 driver, we expressed *GlyP* transgenes in long-lived *dilp2* mutant adults. With addition of RU486, we induced variants of *GlyP* to determine if any modulate the longevity benefit of the *dilp2* mutation. RU486 treatment slightly extended lifespan in controls harboring UAS-*GlyP* transgenes while lacking Gal4 transgenes. To identify mortality effects dependent on *GlyP* independent of side effects from RU486, life table data were fit to Gompertz survival models by accelerated failure analysis. RU significantly affected the scale parameter (λ, frailty) but not the shape parameter (γ, slope). To account for this effect on mortality in the experimental genotypes, we subtracted the estimated scale coefficient associated with RU alone from the scale parameter estimated in Gal4/UAS genotypes given RU. Using these adjusted scale parameters and the estimated shape parameters, we recalculated life tables, survival plots and inferences on mortality. After adjusting for side effects of RU alone, survival does not differ between *dilp2* mutant controls (no RU486) and *dilp2* mutants overexpressing wild-type (Figure 5A); S15D (Figure 5C) *GlyP* (with RU486). As predicted if *dilp2* affects lifespan through control of *GlyP*, longevity is decreased in *dilp2* mutants when we overexpress S15A *GlyP* (Figure 5B). Noting that wild-type and constitutively active *GlyP* extend longevity in adults with wild-type *dilp2*, our genetic interaction analysis verifies that *dilp2* and *GlyP* modulate longevity through a common pathway: *GlyP* activation and phosphorylation at Ser15 is required for mutants of *dilp2* to extend lifespan.

## Discussion

Insulin/IGF signaling variously affects animal cells, tissues, and systemic phenotypes through the action of related ligands, similar receptors and shared downstream components (9, 66). Insulin/IGF signaling is notably complex in *Drosophila* where there are seven insulin-like ligands that interact with a single tyrosine kinase insulin-like receptor, and one relaxin-like ligand associated with a G-protein-coupled receptor (10, 49, 67). Here, we studied how related insulin-like ligands, represented by DILP2 and DILP5, produce distinct phenotypes while signaling through a common tyrosine kinase receptor. The functions of several DILPs have been described by genetic and cellular studies. DILPs have distinct gene expression patterns across development, life stage and tissues [reviewed in Ref. (31)]. The spatiotemporal diversity of DILP expression provides one potential mechanism by which these ligands differentially control phenotypes despite sharing a common receptor. Nevertheless, some DILPs are expressed at the same time and place, such as DILP2, DILP3, and DILP5 in adult IPCs (30), or DILP4 and DILP7 in embryos (1). It seems likely, therefore, that insulin/IGF receptors themselves can differentially signal in response to specific ligands.

Insulin ligand sequence and structure may confer this proposed signaling bias at InR. Primary amino acid sequence varies among DILPs. Some insight on that variation can be gained by noting how DILPs differ from human insulin and IGF. DILPs and insulin/IGF differ at residues thought to be required for the ligands to bind the receptor (36, 68) at insulin binding site 1: Ser B9, Val B10, Tyr B16, and Asn A21; at insulin binding site 2: His B10, Thr A8, and Ile A10. Insulin His B10 is especially interesting because this residue is substituted with Asp to create the fast-acting synthetic insulin analog X10, and because it plays a role in the storage of insulin as hexamers in β-cells (27). The B chain of DILP5, insulin, and IGF contains C-terminal phenylalanines and large aromatic residues, but DILP2 lacks these residues. The phenylalanines and large aromatic residues function in insulin dimerization, negative cooperativity and receptor interaction (69), and their mutation produces diabetes (at human insulin Phe 24 and Phe25, the “insulin Los Angeles” and “insulin Chicago” syndromes) (70, 71). Overall, DILP2 and DILP5 vary in many amino acid residues that could potentially affect receptor binding, and thus differentially modulate receptor conformation and auto-activation. Independent of receptor interactions, DILPs bind circulating factors including acid-labile subunit (dALS), secreted decoy of InR, and IMP-L2 (72–74) which might differentially affect DILP bioavailability and signaling output.

Using synthetic DILP2 and recombinant DILP5, we studied how these related ligands affect cell signaling in a controlled, simplified system: *in vitro* stimulation of *Drosophila* S2 cells. Our preparations of DILP2 and DILP5 ligand have equal potency measured by their ability to induce pAkt in a dose-dependent manner. IIS ligands regulate many cellular functions through control of FOXO transcription factors, so we sought to differentiate these ligands by their potentially unique profiles of induced and repressed mRNA. Based on RNA-Seq and quantitative RT-PCR, DILP2 and DILP5 produce strikingly similar changes in gene expression, where differences are marked by the quantity of transcripts, not their identity. FOXO thus appears to be similarly regulated by DILP2 and DILP5. Mammalian insulin and IGFs provide a precedent for this outcome, where these functionally distinct yet similar hormones produce remarkably concordant transcriptional profiles (18, 19). Naturally, insulin and IGF also operate within cells through non-genomic pathways such as repressing glycogen synthesis and GSK3β, inducing glucose transport through GLUT4 and altering protein translation (58). To study such alternatives, we compared how DILP2 and DILP5 affect the kinetics and patterns of protein phosphorylation.

We began with Western analysis of InR and Akt phosphorylation across a time course, using time points previously studied in mammalian insulin/IGF signaling and in *Drosophila* cells stimulated with human insulin (58, 59). DILP2 and DILP5 stimulate prolonged phosphorylation of InR across the assay period, as seen in rat glial cells when IGF-1 simulates IGF-1R (75, 76). On the other hand, DILP2 induces transient Akt phosphorylation that peaks at 3 min, while DILP5 stimulates prolonged, elevated Akt phosphorylation over the tested 60-min time course. Transient versus prolonged kinetics of Akt phosphorylation resembles the differences seen in mammalian tyrosine kinase receptor signaling where ligand-specific receptor-binding kinetics produce “shout” versus “whisper” transduction dynamics (56) with respective associated metabolic and mitogenic potential (26, 27). In mammalian cell model systems, various insulin and insulin analogs likewise can stimulate transient or sustained dynamics of Akt phosphorylation (25).

The dynamics of IR activation has been modeled based on receptor-binding kinetics induced by specific ligands (23, 24). DILP2 and DILP5 may affect receptor off-rates in distinct due to differences in amino acid sequences that induce unique conformational changes. Conformational changes in receptor extracellular and intracellular domains may subsequently alter receptor auto-activation, trafficking, and turnover (77). Models of mammalian receptor tyrosine kinases propose that different signals may result from trafficking of the receptor when it is ubiquitinated and degraded, recycled to the cell surface, or continuously activated in endosomes (56, 57, 78). Each mechanism may impact how the insulin ligands affect receptor interactions with substrate and adaptor proteins. *Drosophila* has a single IRS *chico*, the homolog of mammalian IRS1–4. In our phosphoproteome analysis, DILP2 and DILP5 similarly phosphorylated Chico Tyr470 and Ser471 (see Table S3 in Supplementary Material), suggesting that these sites do not mediate the distinct impact of the ligands on pAkt dynamics. Many additional adaptor proteins are known for mammalian IIS ligands (79) and several are described for *Drosophila* including Dock, Shc, lnk, and daughter of sevenless (DOS), the homolog of mammalian Gab1 (80). Interestingly, we found that DILP2 increased phosphorylation of the DOS peptide at Thr515 and Thr518 to a greater extent than DILP5. DOS/Gab1 is a scaffold for receptor tyrosine kinase signaling that integrates sevenless signaling, PI3K, and MAPK (81). Future studies may determine if DOS mediates how DILP2 and DILP5 differentially affect the kinetics of pAkt.

Most phosphopeptides affected by DILP stimulation are downstream of the immediate InR substrates. Notably, DILP2, but not DILP5, stimulated dephosphorylation of Cindr, the *Drosophila* homolog of human CIN85, which is associated with endocytosis and clathrin in mammalian cells (82). Moreover, DILP2 dephosphorylated CG6454, a likely homolog of human C2CD5 that interacts with clathrin to regulate GLUT4 translocation in adipocytes (83, 84). Interestingly, inhibition of clathrin-mediated endocytosis can alter insulin-stimulated PI3K activity and Shc and MAPK phosphorylation (78). Thus, DILP2 in contrast to DILP5 may uniquely regulate dynamics of InR endocytosis, GLUT4 membrane translocation, and cytoskeletal structures.

Among other stimulated proteins, glycogen phosphorylase (GlyP) is dephosphorylated by DILP2 but not by DILP5. To evaluate if DILP2 affects GlyP function in S2 cells, we applied a GlyP enzymatic activity assay for insects, adapting techniques from mammalian biology (48). DILP2 treatment decreases GlyP activity, as predicted from the phosphoproteomic data where DILP2 stimulation dephosphorylated GlyP at Ser15. DILP5 stimulation does not alter GlyP activity, in accordance with the phosphoproteomic data where DILP5 did not alter GlyP Ser15. We also validated that DILP2 uniquely regulates GlyP activity *in vivo*. Adult *dilp2* mutant flies, which are long-lived, have elevated GlyP enzymatic activity relative to wild-type flies, while activity is slightly decreased in *dilp5* mutants. Since DILP5 alone does not reduce GlyP Ser15 phosphorylation, the *dilp5* mutation may decrease GlyP activity indirectly because loss of *dilp5* increases *dilp2* expression (data not shown).

*dilp2* mutants could in part extend lifespan by its non-genomic control of this glycogen catabolism enzyme, given that DILP2 regulates GlyP Ser15 phosphorylation and that *dilp2* mutants have increased lifespan and GlyP activity. We find that overexpression of GlyP in all tissues modestly (12%) but significantly extends lifespan. However, Bai et al. previously found that depletion of GlyP by RNAi in all tissues was sufficient to extend lifespan (53), while in yeast, loss of GlyP shortens chronological lifespan (63). Although GlyP modulates glycogenolysis in many tissues, it is unknown whether glycogen levels may affect aging. Longevity is not correlated with stored glycogen among yeast mutants lacking various genes involved in glucose and glycogen metabolism (64). Human cultured muscle cells overexpressing glycogen phosphorylase had decreased glycogen stores, but also display improved metabolic homeostasis through increased glycogen turnover, elevated lipid storage, and enhanced glucose uptake (85). Thus, GlyP overexpression may enhance overall metabolic homeostasis.

Aside from the impact of GlyP on stored glycogen, Favre et al. suggested that *GlyP* may regulate aging through AMP-activated protein kinase (AMPK) and oxidative stress resistance (64). The β-subunit of AMPK has a glycogen-binding domain that acts as a glycogen sensor. Allosteric regulation of this domain affects AMPK phosphorylation and subsequent localization and activity (86). Here, we found increased phosphorylation of the AMPK β-subunit (see Table S4 in Supplementary Material), by DILP2 but not by DILP5 suggesting that activation of fly GlyP may affect aging through secondary effects of altered glycogen flux, rather than simply through glycogenolysis and glucose production. Notably, Gusarov et al. recently demonstrated in *C. elegans* that glycogen modulates longevity by modulating AMPK, superoxide dismutase and oxidative stress resistance, while glycogen phosphorylase deficient worms are short-lived (63). In overwintering insects, glycogen is stored in preparation for diapause and is broken down by glycogen phosphorylase during diapause, a life history state associated with slow to negligible aging (87, 88). Remarkably, GlyP expression is increased during *Drosophila* diapause (89). Therefore, GlyP is situated at the center of a network that regulates energetics, metabolic homeostasis and stress responses that associate with persistence and longevity.

While mammalian glycogen phosphorylase can be activated by glucagon and protein kinase A (PKA) (90), how insulin inversely represses glycogen phosphorylase is not completely understood. Mammalian insulin is proposed to suppress glycogen phosphorylase by activating protein phosphatase 1 (PP1) to dephosphorylate GlyP Ser15 [reviewed in Ref. (90)]. As well, AMP allosterically enhances glycogen phosphorylase activity while glucose represses its activity. When glucose binds to glycogen phosphorylase, the enzyme allows PP1 to dephosphorylate Ser15 (90). Finally, insulin decreases cellular cAMP levels, which represses PKA. Repressed PKA limits phosphorylase kinase activity that otherwise phosphorylates Ser15 on glycogen phosphorylase. DILP2 may modulate *Drosophila* GlyP through each of these mechanisms whereas DILP5 should not. Based on this contrast, we note that the unique control of Cindr and CG6454 phosphorylation by DILP2 indicates potential roles for glucose uptake in cells as possible mechanisms by which DILP2 regulates GlyP dephosphorylation. This conclusion is consistent with the association of *dilp2* with glucose metabolism compared with the association of *dilp5* with protein metabolism (8, 30).

Studies in mammalian insulin/IGF signaling strive to fully understand how these similar ligands can activate varied signaling pathways through similar receptors to produce different phenotypes (19, 79). Furthermore, in humans, insulin and IGF can activate one another’s receptors, as well as IR/IGFR hybrid receptors (9). Consequently, insulin analogs for diabetes treatment can increase mitogenicity and risk of cancer, while manipulating IGF in the treatment of cancer can disrupt glucose homeostasis (91). We demonstrate that DILP2 and DILP5 regulate many parallel cell signaling events, but also uniquely control particular cellular processes to affect distinct organismal physiology. Remarkably, DILP2 uniquely represses GlyP activity, and activation of this enzyme is required for *dilp2* mutants to fully extend longevity. Overall, we demonstrate that two related insulin-like ligands have the capacity to regulate unique traits through a single receptor. We propose that *Drosophila* DILPs bind InR with different kinetics to cause distinct conformational changes in the receptor and effector proteins, and this subsequently produces different signaling output. An explicit comparison of DILP2 and DILP5 receptor-binding kinetics and structures bound to InR will advance how we understand the mechanisms of insulin signaling bias.

## Data Availability Statement

The RNA-Seq datasets generated and analyzed in this study can be found in the Gene Expression Omnibus submission GSE111560. The phosphoproteomic datasets generated and analyzed in this study have been deposited in the ProteomeXchange Consortium (http://proteomecentral.proteomexchange.org) *via* the PRIDE partner repository in the dataset submission PXD009240.

## Author Contributions

SP and MT designed the experiments, interpreted results, and wrote the manuscript; SP and GK conducted the experiments; SP analyzed the data; MT, JW, and PM conceived the research; all the authors revised the manuscript.

## Conflict of Interest Statement

PM is an external consultant to the Department of Stem Cell Research at Novo Nordisk A/S, 2760 Måløv, Denmark. The remaining authors declare that the research was conducted in the absence of any commercial or financial relationships that could be construed as a potential conflict of interest.

## Supplementary Material

Figure S1 DILP2 and DILP5 induce similar gene expression by RNA-Seq. **(A)** Venn Diagram of differentially expressed genes by DILP2 or DILP5 stimulation. **(B)** PCA plot reveals separation of DILP stimulation from controls and slight separation of DILP2 from DILP5. Volcano plots reveal that DILP2 **(C)** and DILP5 **(D)** both stimulate gene expression changes with strong significance but relatively small log2 fold changes. **(E)** Two differentially expressed genes unique to DILP2 stimulation from RNA-Seq were verified by q-RT-PCR from separate biological samples, *n* = 3, **p* < 0.05, ***p* < 0.01.

Figure S2 DILP2 and DILP5 stimulate changes in phosphopeptide abundance by phosphoproteomics. **(A–F)** Volcano plots for phosphopeptides identified in DILP2 **(A–C)** or DILP5 **(D–F)** stimulation at the indicated time point. All conditions reveal large log2 fold change values (Log2(FC)) with small significance (Log(Q value)). **(G–L)** Inflection points were calculated from all phosphopeptide log2 fold changes for each stimulation condition. Dotted lines represent the inflection point y-values selected as cut-offs for fold-change thresholds.
