# Supplementary figures and images for: Drosophila Insulin-Like Peptides DILP2 and DILP5 Differentially Stimulate Cell Signaling and Glycogen Phosphorylase to Regulate Longevity
Source: Front Endocrinol (Lausanne). 2018 May 28;9:245. doi: 10.3389/fendo.2018.00245 (PMC5985746; doi:10.3389/fendo.2018.00245)

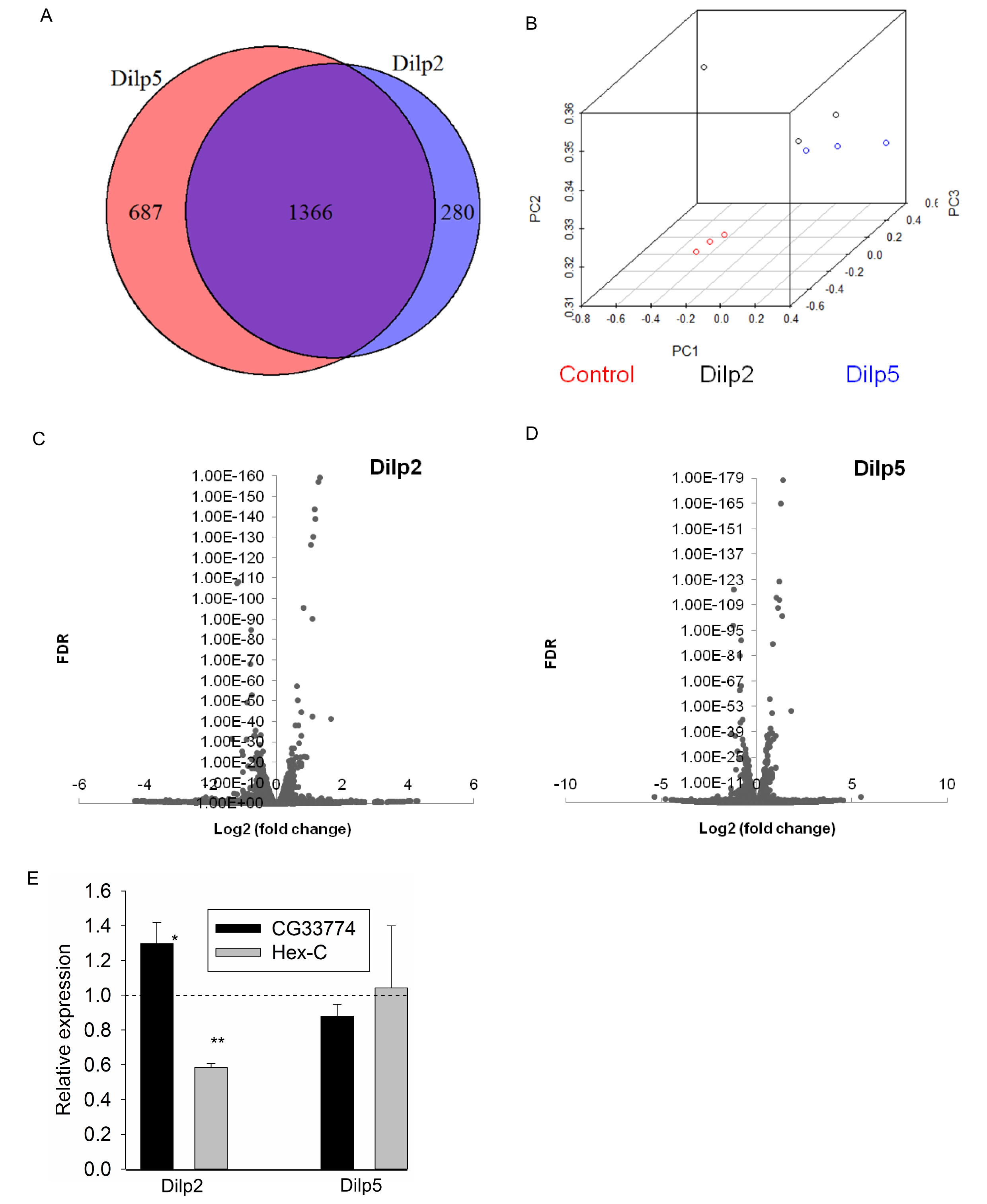

Supplement: Figure S1 — DILP2 and DILP5 induce similar gene expression by RNA-Seq. (A) Venn Diagram of differentially expressed genes by DILP2 or DILP5 stimulation. (B) PCA plot reveals separation of DILP stimulation from controls and slight separation of DILP2 from DILP5. Volcano plots reveal that DILP2 (C) and DILP5 (D) both stimulate gene expression changes with strong significance but relatively small log2 fold changes. (E) Two differentially expressed genes unique to DILP2 stimulation from RNA-Seq were verified by q-RT-PCR from separate biological samples, n = 3, *p < 0.05, **p < 0.01. [file image_1.tif]

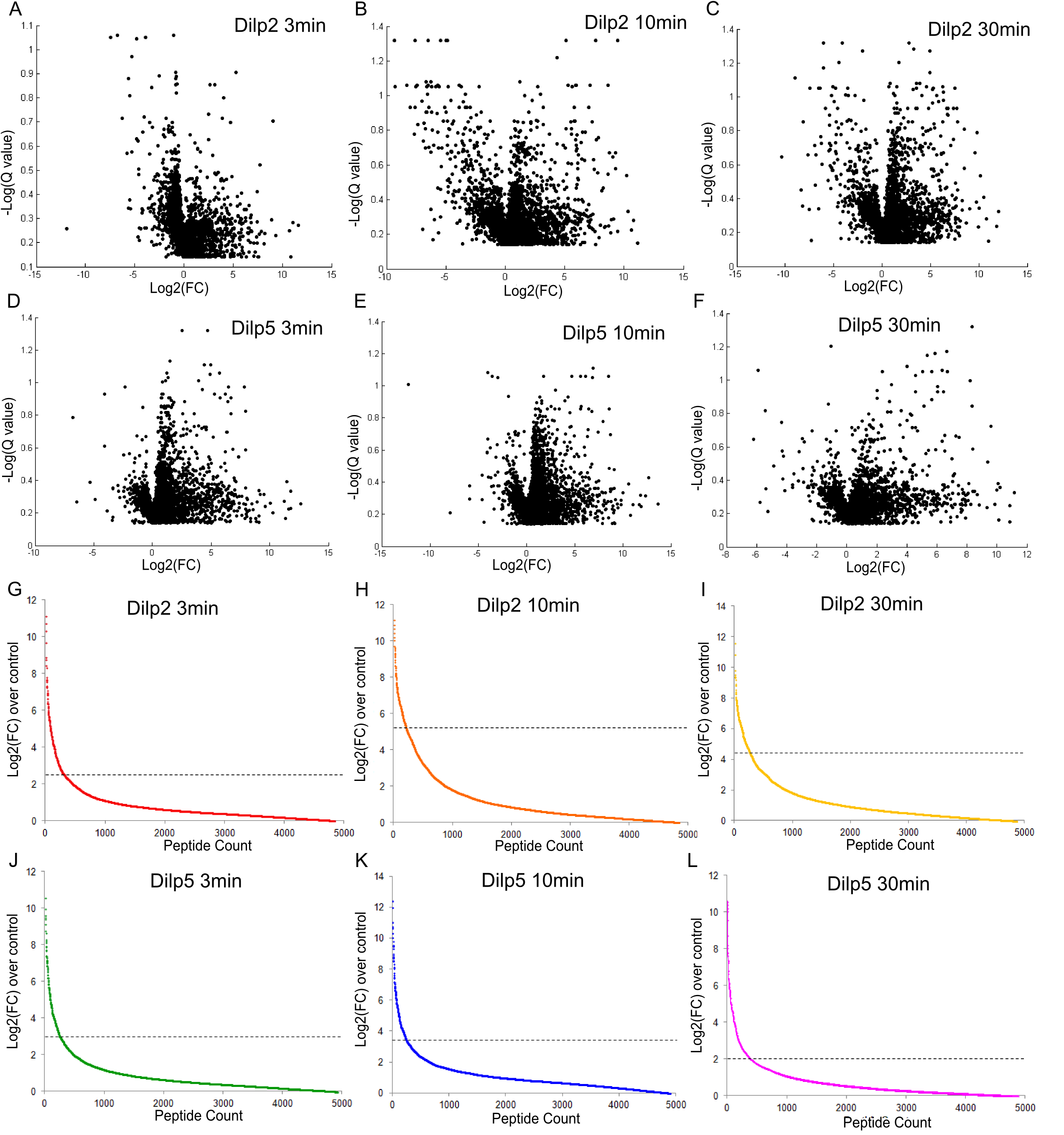

Supplement: Figure S2 — DILP2 and DILP5 stimulate changes in phosphopeptide abundance by phosphoproteomics. (A–F) Volcano plots for phosphopeptides identified in DILP2 (A–C) or DILP5 (D–F) stimulation at the indicated time point. All conditions reveal large log2 fold change values (Log2(FC)) with small significance (Log(Q value)). (G–L) Inflection points were calculated from all phosphopeptide log2 fold changes for each stimulation condition. Dotted lines represent the inflection point y-values selected as cut-offs for fold-change thresholds. [file image_2.tif]
